# Preventive home visits postpone mortality – a controlled trial with time-limited results

**DOI:** 10.1186/1471-2458-6-220

**Published:** 2006-08-31

**Authors:** Klas-Göran Sahlen, Lars Dahlgren, Britt Mari Hellner, Hans Stenlund, Lars Lindholm

**Affiliations:** 1 Epidemiology and Public Health, Umeå University, S-901 85 Umeå, Sweden; 2 IMS, Institute for evidence-based social work practice, The National Board of Health and Welfare, S-106 30 Stockholm, Sweden

## Abstract

**Background:**

There is a debate on whether preventive home visits to older people have any impact. This study was undertaken to investigate whether preventive home visits by professional health workers to older persons can postpone mortality in a Swedish context.

**Method:**

A controlled trial in a small community in the north of Sweden.

Participants are healthy pensioners aged 75 years and over. 196 pensioners were selected as the intervention group and 346 as the control group. The intervention, two visits per year, lasted two years.

**Results:**

During the intervention, mortality was 27 per 1000 in the intervention group and 48 per 1000 in the control group. The incidence rate ratio for the control group IR_2000–2001 _was 1,79 (95%CI = 0,94–3,40). Analysing the data with an "on treatment approach" gave a significant result, 2,31 (95%CI = 1,07–5,02) After the trial the difference between the groups disappeared.

**Conclusion:**

Preventive home visits in a healthy older population can postpone mortality in a Swedish context if they are carried out by professional health-workers in a structured way. When the home visit programme ended the effect on mortality disappeared. These findings are dependent on contextual factors that make it difficult to form general policy recommendations.

## Background

Can health professionals postpone mortality in old age with preventive home visits? According to this Swedish trial the answer is yes. All over the world the number of elderly people is increasing. In 2002 the world had approximately 440 million people aged 65 years and over (7,1%). During the coming 25 years the number of people over 65 years is expected to double [1]. In the least developed countries of the world the ageing population is growing at an even faster rate [2]. With a higher proportion of older people worldwide it is reasonable to expect an increase in the prevalence of illness [3] and increasing global demands on resources for health care. To reduce poor health in old age, methods such as screening, health check-ups and physical training programmes are used in OECD countries. One possible strategy to improve health among older people is preventive home visiting programmes (PHV). In this article we use the definition of PHV made by van Haastregt [4] as "visits to independently living elderly people, which are aimed at multidimensional medical, functional, psychosocial, and environmental evaluation of their problems and resources." The results are specific recommendations and suggestions aimed at reducing observed problems and preventing new ones.

More than twenty years ago a Danish trial, the Roedovre project [5], showed that home visits to old people reduced utilisation of care. Significantly fewer admissions to hospital and emergency medical calls were registered in an intervention group compared to a control group. Since this study, several researchers [4,6-9] have produced varying results. Some [5,7] argue that home visits can postpone functional decline or mortality. Others [9] indicate that multidimensional programmes are inefficient when the target group is older people at risk.

The effectiveness of PHV has been questioned in a systematic review, with the authors' recommendation to discount these visits if the effectiveness was not improved [4]. A meta-analysis based on 28 controlled trials concluded that there was potential to reduce mortality risk and improve functional ability through PHV[10]. There appears to be conflicting results with respect to PHV and mortality, possibly due to the heterogeneity of the studies. If the comparison is restricted to similar trials with respect to aim, age, staff and follow-ups, we find that three studies present similar conclusions concerning mortality. The Danish and UK trials [5,11] showed significant reductions in mortality, while the Netherlands trial [12] showed a non-significant reduction (17% in the control compared with 14% in the intervention group).

In Australia and Denmark there is legislation governing preventive home visits. In 1999 the Swedish Government invited local authorities and primary care districts to apply for financial support to develop models for preventive home visits in Sweden. Twenty one projects all over Sweden received grants, among them Nordmaling, a rural community with less than 8000 citizens, in the north of Sweden. The project in Nordmaling showed that preventive home visits, targeting healthy people 75 years and older, had a positive effect on morbidity, utilisation of care and quality of life[13]. The objective of this study was to investigate whether PHV also postponed mortality.

## Method

### The home visit trial

The preventive home visits in Nordmaling were performed during 2000 and 2001. When planning for the project local pensioner associations were important collaborators. They influenced the content of the visits, the employment of staff, and ethical aspects of carrying out the visits as a controlled trial. One nurse and one care manager were employed to carry out the project. Half of the pensioners were assigned to each visitor. The nurse and the care manager followed their "own" pensioners for the duration of the programme, making visits to each pensioner's home. Since the two visitors were trained in different occupational paradigms, it was decided that they should continuously share knowledge and experiences from the visits. The purpose was to bring the two visitors closer with respect to their different backgrounds and to give the pensioners similar advice and information.

Each pensioner was visited four times, once every six months. Each visit lasted for 1,5 to 3 hours and followed a structured program. According to the program general information about physical activity, symptoms of common diseases of the elderly, influenza vaccination, diet, and awareness of risks for fall injuries and what to do to avoid them, were given. A questionnaire (see Additional file 1) was completed every visit and different dimensions of self reported health, functional ability, well-being and social networks were recorded. The respondents usually judged their situation on a scale with four alternatives. The questionnaire functioned as an interview guide as well as an evaluation instrument.

#### All visits

A multi dimensional questionnaire was used. A structure of main themes was used but this did not prevent researchers from taking into account the individual situation of each pensioner. The maintenance of the social network as a tool for better health was stressed.

#### First visit

Focus on physical activity and discussion about risks in the home

#### Second visit

Focus on preventing falls, information about influenza vaccine and examples of activities in society suitable for the person

#### Third visit

Focus on healthy food and information about diabetes

#### Fourth visit

Focus on knowledge about home help, long-term care, access to health- and dental care.

No regular information from the home-visitor to the primary health care or the long-term elderly care provider was made. Occasionally, when the senior was in need of some kind of assistance, the home-visitor followed-up before the next visit by telephone, or extracted a promise from the senior to contact a primary health care centre. The visitor assessed each individual's situation and acted accordingly. Thus a person in need of assistive devices or drug regime review, for example, would be given information on available solutions and followed up. Other examples of recommendations include to start the morning with exercises in bed to reduce the risk of falling caused by a drop in blood pressure, if that was a problem, or to participate in suitable social activities or physical exercises, if that was judged to be of value.

### Study participants

**Figure 1:**
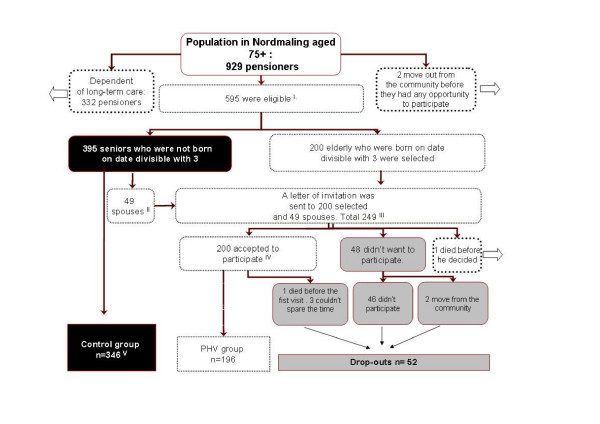
Description of the studied population.

**Figure 2:**
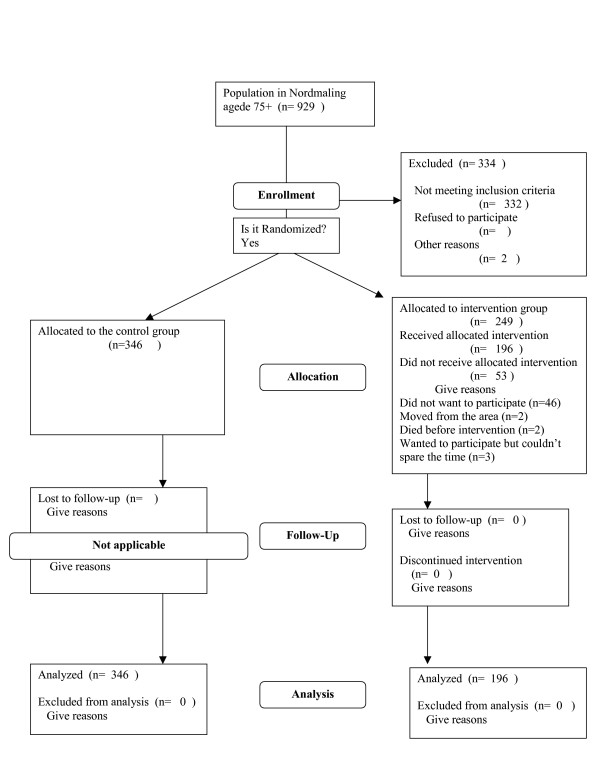
The Consort E-Flowchart.

All inhabitants in Nordmaling, 75 years or older, and living independently without any home help or home-nursing care were eligible for the trial – in total 595 persons (Fig 1. ^I^). Those whose birth date was divisible by three were assigned to the intervention (i.e. born on the 3rd, 6th, 9th etc. of any month). The remaining participants were assigned to the control group. In the intervention group, 49 people (Fig 1. ^II^) were cohabiting with participants initially assigned to the control group. To avoid contamination of the control group, these individuals were also assigned to the intervention group if they were over 75 years and without home help. Thus, 249 pensioners formed the intervention group and (Fig 1. ^III^) were sent a letter of invitation to the trial. 200 individuals accepted the home visits (Fig 1. ^IV^) but one died and three couldn't make the time, hence there were 196 who got the visits. The remainder of the population, 346 seniors (Fig 1. ^V^), formed the control group (Fig 2). Seniors in the control group got no visits. They were not informed of the belonging to any group in this trial. Anyone invited to the trial that moved out of the municipality during the trial were counted in the drop-out group.

A comprehensive analysis of the drop-out group was conducted. An important part of this analysis was a structured telephone interview aimed at gathering information as to why these individuals declined to participate.

### Statistical methods and analyses

Baseline data were collected from the Swedish National registration and the registration of patients in hospital care made by the county council in Västerbotten. All mortality data came from the Swedish national registration. The Cohort software was used to calculate the time at risk for each individual from the date when they entered the study. (The software is developed by the Department of Public Health and Clinical Medicine, Epidemiology and Public Health Sciences, Umeå University, and it can be obtained as freeware from the author.) Incidence rate ratios, with the PHV group as reference group, were calculated with a confidence interval (95% CI). Calculations were made both as "intention to treat" (ITT) and as "on treatment" analyses. The trial period was compared with a post-trial period starting in January 2002 and ending in October 2004. Subgroups, based on gender and cohabitation, were analysed.

### Ethical approval

Informed consent was obtained from every person in the intervention group. All participants were also invited to a presentation of the preliminary results when the trial had ended. Permission from the Research Ethics Committee at Umeå University was obtained for this study (dnr 02–445).

## Results

The groups in the trial were similar with respect to gender, age, and not having been hospitalised during 1999. The mean age was 79 years and there were more woman than men (Table 1). In the intervention group the proportion of cohabitants was higher because 48 spouses initially randomized to the control group were included in the intervention group.

During the two years of intervention mortality was 27 per 1000 years in the intervention group and 48 per 1000 years in the control group. The incidence rate ratio IR_2000–2001_, using the control group as reference, was 1,79 (95%CI = 0,94–3,40) (Table 2). Analysing the data with an "on treatment" approach, gave a significant result, 2,31 (95%CI = 1,07–5,02) (Table 3). During the follow-up period (2002–2004) the mortality increased in both groups but the difference between the groups disappeared. The mortality in both groups was 60 per 1000 years, Subgroup differences during the intervention period, although not statistically significant, are interesting. For women the "on treatment" analysis gave an IR _2000–2001 _= 3,95 (95 % CI = 0,89–17,40) and for persons living alone an IR _2000–2001 _= 6,27(95% CI = 0,83–47,26).

## Discussion

**Figure 3:**
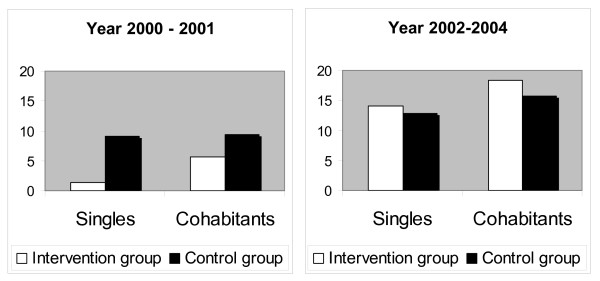
Distribution of mortality during and after the trial. (%).

The results from Nordmaling indicate that mortality is influenced by preventive home visits. The result for the intervention period is significant when an "on treatment" analysis is used despite the relatively small groups. The result also shows that both men and woman gain from PHV, even if female mortality in general is lower. In terms of mortality there is a tendency for single persons to gain more from PHV than cohabitants (Fig 3.). However the positive effect is there only as long as PHV are ongoing. Is the effect on mortality time-limited or was compliance to the recommendations lower when the programme of visits ended? We cannot answer that question. We know that many seniors described that the visits gave a sense of security, a feeling of being important and they reported improved self-rated health along the path of visits. That could partly explain the difference in mortality patterns. It has been shown that self-rated health is important to maintaining good health[14].

The trial in Nordmaling was one of 21 different PHV projects in Sweden and the results concerning health and well-being from the other trials were also positive [15,16], but the effect of PHV on mortality has not been measured elsewhere in Sweden. When conducting a trial in a small community it is important to get public support for the idea that only some community members will potentially benefit from the intervention. In this trial, therefore, the local pensioners' associations were invited to discuss the possibility of all people aged 75+ years receiving PHV in the future. The possibility of expanding the home visit programme to all people aged 75+ years in the community, should the trial produce positive results, was also discussed with representatives of the municipality. The pensioners' associations also became active partners in the management of the trial.

All participants in the trial used regular health care. Health care providers in primary and long-term facilities were blinded to the trial, as far as possible. There were no differences in access to health care or access to other activities in society between the visited and not-visited groups. However there might be a difference in knowledge.

The intervention group was established for this trial alone and was not used for any additional studies or interventions, therefore there was no contamination from other activities.

The design and analysis of this kind of "real-life" intervention is not self-evident. To reduce contamination between the intervention group and the control group, the spouses of pensioners randomized to the intervention group were also invited to participate. As a consequence the proportion of cohabitants is larger in the intervention group than in the control group. This difference gives rise to opposite effects. The death rates in the intervention group during the study period can be expected to be lower because cohabitant living is normally a protective factor. On the other hand, our data reveals that the interventions have had a smaller effect among cohabitants. The effects may cancel each other out because the result, when excluding the spouses allocated to the intervention group, due to family affiliation and not due to birth-date did not change. Furthermore, it is unrealistic to assume that all members in the control group had absolutely no exposure to the intervention [17]. Neighbours talk and friends share experiences. This indicates that the data presented may therefore be an underestimate.

One in five potential participants in the study declined the invitation to participate (Figure 1). An effort was made to telephone all 46 persons who refused to participate and still lived in the community. Five individuals refused to give any additional information and 16 individuals did not answer the call. Thirteen people stated that they had no need for a visit and four individuals gave different medical reasons for not participating. The remainder stressed different aspects of personal autonomy such as "I manage by myself" or "I want to have privacy". In the drop-out group the proportion of youngest old (75–80 years) is higher than in the other groups. Admission to hospital before the trial was less common in the drop-out group, (Table 1) indicating that it is reasonable to expect a lower mortality rate in the drop-out group.

Considering the circumstances described above, the best mode of analysis is not obvious. The allocation of spouses as well as drop-outs has probably introduced bias. One important reason to use an "intention-to-treat" (ITT) analysis is that this analysis reflects how PHV, in this case, will perform in a population and takes care of the selection bias introduced by drop-outs. However, the inclusion criteria used are very general and the natural selection process is possibly more appropriate. The intervention is offered to the all people 75 years and older living independently, and those feeling quite healthy are likely to reject the offer and postpone participation for some years. Taking all into account, we have in this article decided to present the results both as "intention to treat" and as "on treatment" analyses. The differences in effect size and significance level between ITT and OT most likely mirrors the above reasoning – when the more healthy drop-outs are included the effect decreases.

Another important question is whether the efficacy in this trial can be transferred to effectiveness in ordinary care activities. It may be that during this trial, the results were influenced by the Hawthorne effect[18]. The pensioners in the intervention group felt that they were specially selected and appreciated being part of the PHV trial. Elton Mayo describes this phenomenon as one possible explanation for positive results. In this intervention it was desirable to create a Hawthorne effect, not as an explanation for the result but as a part of the intervention itself. It was considered beneficial for the participants to feel that they were appreciated and had the potential to remain healthy and secure. It is important for old people to be appreciated [19] as this can influence self-rated health which in itself can have an impact on health outcomes [20]. This trial demonstrates the advantages of a structured home-visit programme, when carried out as a part of the ordinary health care organisation with well educated and motivated staff.

Stuck et al conclude [6] that the number of admissions to nursing homes is related to the number of home visits with no less than five visits required to produce a beneficial effect. The mortality rate, however, does not seem to be affected. Van Haastregt et al find it unsuitable to use a meta analysis approach [4], and show how different settings influence the conclusions drawn. A recently published report from WHO [21] concludes that PHV can reduce mortality, prevent nursing home admissions and has the potential to be cost effective. Despite these conclusions the authors are very cautious regarding general policy considerations: "This review does not provide evidence for stopping existing home-visiting programmes, but further research is required... prior to implementing new programmes."

Seldom is the content of regular primary health care and long-time care for older people described when PHV trials have been published. This is important if the control group used as the reference uses the same regular services as the intervention group. All people in Sweden have good access to medical treatment, however preventive activities for old people have been withdrawn over the last decade due to lack of resources and there has been a marked redistribution of resources to individuals with comprehensive care needs[22]. This implies that access to preventive health care and long-term care for older persons with fairly good health is less than several years ago. A change in regular care available to the control-group, may alter the result of the intervention, e.g. measured as incidence rate ratio. It is therefore reasonable to state that a home visit programme in Sweden is more valuable today than it would have been a decade ago. In other countries the situation may be different. This trial shows that PHV is favourable in the Swedish context but that it is inappropriate to generalize these findings to other countries and other contexts. While this makes it difficult to formulate general recommendations, we believe that this trial provides sufficient evidence for implementing structured PHV programmes in the Swedish context.

## Conclusion

Preventive home-visits can postpone mortality in a healthy older population but the mortality effect can only be shown during the intervention period. This trial demonstrates the advantages of a structured home-visit programme, when carried out with well educated and motivated staff.

## Competing interests

The author(s) declare that they have no competing interests.

## Authors' contributions

KGS conceived and carried out the preventive home visits study, participated in the discussion of the design, drafted the manuscript and in collaboration with HS did the statistical analysis. HS has also contributed in the result discussion. LD and BMH have contributed in the discussion of the design and have made important contribution to the article. BMH has also constructed the questionnaire (Additional file 1). LL participated in the design of the article and contributed to finalizing the result and discussion parts. All authors have read and approved the final manuscript.

## Pre-publication history

The pre-publication history for this paper can be accessed here:



## Supplementary Material

Additional file 1 **Preventive Home Visits **– The questionnaire. A translation, from Swedish to English, of the used questionnaire. Click here for file

## Figures and Tables

**Table 1:** Characteristics of the three groups

	***Intervention group n = 196***	***Control group n = 346***	***Drop-out group n = 52***	***p-value***
Women (%)	107 (54,6)	196 (56,5)	24 (46,2)	0,455
Mean age, year (± SD)	79,7 (± 3,91)	79,8 (± 4,30)	77,7 (± 2,95)	0,009
Proportion 75 – 80 years (%)	109 (55,6)	206 (59,5)	41 (78,8)	0,000
Cohabitants (%)	125 (63,8)	159 (45,8)	no info	0,000
No hospital visit, 1999(%)	159 (81,1)	274 (79,2)	44 (84,6)	0,849

**Table 2:** Mortality in the intervention group compared with the controls – intention to treat

	**Year**	**n**	**Risk time (year)**	**Death**	**Death/1000 years**	**Incident rate ratio**	**CI (95%)**
**All participants**							
Intervention group	2000–2001	248	483	13	27	1	
Control group	2000–2001	346	666	32	48	1,79	0,94–3,40
Intervention group	2002–2004	235	616	37	60	1	
Control group	2002–2004	314	831	50	60	1,00	0,66–1,53
**Woman**							
Intervention group	2000–2001	131	259	3	12	1	
Control group	2000–2001	195	377	14	37	3,21	0,92–11,16
**Men**							
Intervention group	2000–2001	117	224	10	44	1	
Control group	2000–2001	151	288	18	63	1,40	0,65–3,03

**Table 3:** Mortality in the intervention group compared with the controls – on treatment

	**Year**	**n**	**Risk time (year)**	**Death**	**Death/1000 years**	**Incident rate ratio**	**CI (95%)**
**All participants**							
Intervention group	2000–2001	196	385	8	21	1	
Control group	2000–2001	346	666	32	48	2,31	1,07–5,02
Intervention group	2002–2004	188	485	32	66	1	
Control group	2002–2004	314	831	50	60	0,91	0,59–1,42
**Living single**							
Intervention group	2000–2001	71	141	1	7	1	
Control group	2000–2001	187	360	16	44	6,27	0,83–47,26
**Cohabitants**							
Intervention group	2000–2001	125	244	7	29	1	
Control group	2000–2001	159	305	16	52	1,89	0,75–4,44
**Woman**							
Intervention group	2000–2001	107	213	2	9	1	
Control group	2000–2001	195	377	14	37	3,95	0,89–17,40
**Men**							
Intervention group	2000–2001	89	172	6	35	1	
Control group	2000–2001	151	288	18	63	1,79	0,71–4,51
