# Supplementary material for: Preventive home visits postpone mortality – a controlled trial with time-limited results
Source: BMC Public Health. 2006 Aug 31;6:220. doi: 10.1186/1471-2458-6-220 (PMC1584405; doi:10.1186/1471-2458-6-220)
Supplement: Additional file 1 — Preventive Home Visits – The questionnaire. A translation, from Swedish to English, of the used questionnaire. [file 1471-2458-6-220-S1.doc]

HS/Äldreenheten/BMH

1999-12-09, reviderat 2002-11-25, English translation 2006-06-26

## Preventive Home Visits

Interview

1. Are you living alone?

1 🞎 Yes If yes:

Since year_________

2 🞎 No If no:

With whom do you cohabit?

1 🞎 Spouse

1 🞎 Child

1 🞎 Sibling

1 🞎 Other

2. Do you manage the following activities without help if needed?

Buying food 1 🞎 Yes 2 🞎 No

Cooking 1 🞎 Yes 2 🞎 No

Laundry 1 🞎 Yes 2 🞎 No

Cleaning 1 🞎 Yes 2 🞎 No

Get up or go to bed 1 🞎 Yes 2 🞎 No

Dress or undress 1 🞎 Yes 2 🞎 No

Use the toilette 1 🞎 Yes 2 🞎 No

Take a bath or shower 1 🞎 Yes 2 🞎 No

3. Do you practise any physical activities, e.g. short walks, biking, housework or gardening?

1 🞎 Never/hardly ever

2 🞎 Less than once a month

3 🞎 Once or twice a month

4 🞎 Several times a month

5 🞎 Several times a week

6 🞎 Daily

4. In your opinion, how is your state of health? Is it..

1 🞎 Very good

2 🞎 Good

3 🞎 Bad

4 🞎 Very bad

5. Do you feel fatigued?

1 🞎 Hardly ever

2 🞎 Sometimes

3 🞎 Often

4 🞎 Most of the time

6. Do you feel low-spirited?

1 🞎 Hardly ever

2 🞎 Sometimes

3 🞎 Often

4 🞎 Most of the time

7. Do you feel uneasiness or anxiety?

1 🞎 Hardly ever

2 🞎 Sometimes

3 🞎 Often

4 🞎 Most of the time

8. Do you feel aches or pains?

1 🞎 Hardly ever

2 🞎 Sometimes

3 🞎 Often

4 🞎 Most of the time

9. How often do you receive visits?

1 🞎 Never/hardly ever

2 🞎 Once a month

3 🞎 Once a week

4 🞎 Daily

10. How often do you visit somebody?

1 🞎 Never/hardly ever

2 🞎 Once a month

3 🞎 Once a week

4 🞎 Daily

11. Are you pleased with your life as it turned out?

1 🞎 Very pleased

2 🞎 Pleased

3 🞎 Displeased

4 🞎 Very displeased

12. Mobility-Tiredness Scale

Score___________

13. Timed get up and go

Score (number of seconds)___________
